# Therapeutic potential of coenzyme Q_10_ in mitochondrial dysfunction during tacrolimus-induced beta cell injury

**DOI:** 10.1038/s41598-019-44475-x

**Published:** 2019-05-29

**Authors:** Kang Luo, Ji Hyun Yu, Yi Quan, Yoo Jin Shin, Kyung Eun Lee, Hong Lim Kim, Eun Jeong Ko, Byung Ha Chung, Sun Woo Lim, Chul Woo Yang

**Affiliations:** 1 Convergent Research Consortium for Immunologic Disease, Seoul St. Mary’s Hospital, The Catholic University of Korea School of Medicine, Seoul, Republic of Korea; 2 Transplant Research Center, The Catholic University of Korea School of Medicine, Seoul, Republic of Korea; 3 Department of Internal Medicine, Seoul St. Mary’s Hospital, The Catholic University of Korea School of Medicine, Seoul, Korea; 4 Advanced Analysis Center, Korea Institute of Science and Technology, Seoul, Korea; 5 Integrative Research Support Center, The Catholic University of Korea School of Medicine, Seoul, Republic of Korea

**Keywords:** Metabolic disorders, Kidney

## Abstract

We previously reported that oxidative stress induced by long-term tacrolimus treatment impairs mitochondrial function in pancreatic beta cells. In this study, we aimed to investigate the therapeutic potential of coenzyme Q_10_, which is known to be a powerful antioxidant, in mitochondrial dysfunction in tacrolimus-induced diabetic rats. In a rat model of tacrolimus-induced diabetes mellitus, coenzyme Q_10_ treatment improved pancreatic beta cell function. The administration of coenzyme Q_10_ improved insulin immunoreactivity within islets, which was accompanied by reductions in oxidative stress and apoptosis. Assessment of the mitochondrial ultrastructure by electron microscopy revealed that coenzyme Q_10_ treatment increased the size, number, and volume of mitochondria, as well as the number of insulin granules compared with that induced by tacrolimus treatment alone. An *in vitro* study using a pancreatic beta cell line showed that tacrolimus treatment increased apoptosis and the production of mitochondrial reactive oxygen species, while cotreatment with coenzyme Q_10_ effectively attenuated these alterations. At the subcellular level, tacrolimus-induced impairment of mitochondrial respiration was significantly improved by coenzyme Q_10_, as evidenced by the increased mitochondrial oxygen consumption and ATP production. Our data indicate that coenzyme Q_10_ plays an important role in reducing tacrolimus-induced oxidative stress and protects the mitochondria in pancreatic beta cells. These findings suggest that supplementation with coenzyme Q_10_ has beneficial effects in tacrolimus-induced diabetes mellitus.

## Introduction

Tacrolimus (Tac), a calcineurin inhibitor (CNI), is the most commonly used immunosuppressant. However, chronic treatment with Tac following kidney transplantation is associated with an increased incidence of new-onset diabetes mellitus (DM) after transplantation (NODAT)^1^. A 5-year follow-up study of patients being treated with Tac after transplantation showed a frequency of DM of 41%^2^. The diabetogenic action of Tac is not understood, but pharmacologic calcineurin inhibition, important in preventing rejection during organ transplantation, is related to post-transplantation beta cell failure^3–10^. Various studies have revealed that this condition is partly related to the toxicity of Tac on beta cells in the pancreas, with oxidative injury playing a central role in Tac-induced beta cell dysfunction^11–13^.

Coenzyme Q_10_ (CoQ_10_) acts as a diffusible electron carrier in the mitochondrial respiratory chain^14^. CoQ_10_ also acts as a powerful antioxidant that removes free radicals, inhibits the initiation and propagation of lipid peroxidation in cellular biomembranes, and aids in the regeneration of α-tocopherol^15,16^. Earlier papers have revealed the beneficial effects of CoQ_10_ in diverse experimental diseases of oxidative tissues^17–23^. Therefore, CoQ_10_ has the potential to preserve cells against Tac-induced oxidative stress and pancreatic beta cell injury.

Recent studies have shown that Tac has a detrimental effect on mitochondrial function in beta cells. Impaired mitochondrial Ca^2+^ uptake, which is mechanistically linked to mitochondrial dysfunction, has been shown to occur following a reduction in the enzymatic activity of cytochrome c oxidase, the incidence of which is increased in DM following Tac treatment^24^. Based on this knowledge, the current study was conducted to investigate whether the concurrent treatment of CoQ_10_ can ameliorate Tac-induced DM by reducing oxidative stress via its anti-oxidative properties. We also evaluated whether the protective effect of CoQ_10_ is associated with the preservation of mitochondrial function, such as preventing impairments in oxygen consumption of mitochondria and ATP production and an increase in reactive oxygen species (ROS) production during Tac treatment.

## Materials and Methods

### Ethics statement

All procedures were performed in strict accordance with the recommendations of the ethical guidelines for animal studies. All experimental animal care protocols were approved by the Animal Care and Use Committee of the Catholic University of Korea (CUMC-2015-0028-02). Animals were sacrificed under xylazine/rompun anesthesia, and every effort was made to minimize animal suffering.

### Experimental rat model

Male Sprague-Dawley (SD) rats (Orient Bio, Seongnamsi, Korea) were provided a 0.05% sodium diet (Research Diets, New Brunswick, NJ, USA), and water ad libitum. After 7 days, the mice were randomly divided into 4 groups (*n* = 8 per group) and administered 1.5 mg/(kg•day,) Tac (Astellas, Ibaraki, Japan) or 1 mL/(kg•day, subcutaneous) vehicle (Vh: olive oil; Sigma-Aldrich, St. Louis, MO, USA) with/without coenzyme Q_10_ (CoQ_10_; 20 mg/kg•day, diluted in olive oil, daily oral gavage; Chong Kun Dang Pharm., Seoul, Korea) for 4 weeks. Route of administration and doses were selected as per those established in earlier reports^25,26^.

### Basic treatment protocol

Animals were pair-feeding and their body weight was monitored. Rats were housed separately in metabolic cages (Tecniplast, Gazzada, Italy) for assessment of water intake and urine volume for 1 day after the 4-week treatment. On the next day, rats were anesthetized, and their blood and tissue were harvested for the following experiments. The Tac concentration in whole-blood was measured using liquid chromatography-tandem mass spectrometry (Abbott Diagnostics, Abbott Park, IL, USA)^27^.

### Pancreatic function and beta cell area in islets

After the 4-week treatment, an intraperitoneal glucose tolerance test (IPGTT) was conducted for measurement of the glucose level. The data obtained in the IPGTT was calculated by trapezoidal estimation for the area under the curve of glucose (AUCg). The fasting insulin level in serum was examined using an ELISA Kit (Millipore Corp). HbA1c was measured in whole blood using Cobas Integra 800 (Roche Instrument Center AG, Rotkreuz, Switzerland). For quantification of the beta cell area, the numbers of beta cells were assessed in each sample using captured images following immunohistochemical staining for insulin (TDI Scope Eye Version 3.6 for Windows; Seoul, Korea). Insulin-positive cells were randomly selected and counted by randomly selecting 20 islets from the eight animals in each group.

### Immunohistochemistry

After processing with the retrieval solution (pH 6.0), methanolic H_2_O_2_, 0.5% of Triton X-100, and 10% of normal serum (Jackson ImmunoResearch, West Grove, PA, USA), the tissue sections were incubated for 1 day at 4 °C with primary antibodies against insulin (18-0067, Invitrogen, Carlsbad, CA, USA), 8-hydroxy-2′-deoxyguanosine (8-OHdG: MOG-100P; JaICA, Shizuoka, Japan), 4-hydroxy hexenal (4-HHE: MHH-030n; JaICA), or Ki67 (ab15580, Abcam, Cambridge, UK) and then with peroxidase-conjugated antibodies (Molecular Probes, Carlsbad, CA, USA). The activity of peroxidase was evaluated using 3,3′-diaminobenzidine (DAB; Vector Laboratories, Burlingame, CA, USA). For double labeling with insulin, the 8-OHdG- and 4-HHE-stained tissue sections were washed with phosphate-buffered saline and then treated with an anti-insulin antibody (Invitrogen) and cyanine 3 (Cy^3^)-labeled antibody (Jackson ImmunoResearch). Stained tissues sections were observed under a Zeiss LSM700 confocal microscope (Carl Zeiss MicroImaging GmbH, Jena, Germany). The number of 8-OHdG- or 4-HHE-labeled cells were determined by measuring approximately 20 randomly chosen non-overlapping islets from the eight animals in each group. Using histogram equalization, the percent positive area was calculated by quantitative analysis under the same intensity (TDI Scope Eye).

### Measurement of beta cell mass

The relative volumes of beta cells were estimated by the point-counting method^28^. The relative beta cell volume was measured by classifying the number of points matching to the insulin-positive region by the number of points matching to the remaining pancreatic area. Cell masses were measured by multiplying the ratios of beta cells by the total pancreatic weight^29^.

### Glucose-stimulated insulin secretion (GSIS) assay

As described earlier, islets were isolated from male SD rats (250–300 g) by digestion using collagenase^30,31^. The islets were pre-incubated in conditioned RPMI 1640 medium at 37 °C for 24 h. The isolated islets were then treated with Tac (1 μg/mL) and CoQ_10_ (10–1000 ng/mL) for 12 h. This was followed by analysis of insulin secretion. The harvested islets were divided into groups of 30 islets and washed with Krebs-Ringer Modified Buffer (KRB), followed by addition of 2.8 mM glucose (basal). After washing with KRB, the islets were incubated with KRB containing 16.7 mM glucose for 1 h. The insulin level in the solution was examined using an ELISA kit (Millipore Corp., St. Charles, MO, USA).

### Measurement of 8-OHdG in serum

The end product of oxidative DNA damage was measured by determining the concentration of 8-OHdG in serum. The 8-OHdG level was evaluated using an ELISA kit (Cell Biolabs, San Diego, CA, USA).

### Terminal deoxynucleotidyl transferase dUTP nick end labeling (TUNEL) assay

The TUNEL assay was performed for the tissue sections using the Apoptosis Detection kit (Millipore Corp.) as per the manufacturer’s instructions. For double labeling with insulin, tissue sections were incubated with an insulin antibody (Invitrogen) followed by incubation with a Cy^3^-labeled antibody (Jackson ImmunoResearch). The double-labeled cells were counted in approximately 20 randomly selected non-overlapping islets per animal of each group.

### Transmission electron microscopy

Electron microscopic inspection was processed as previously described^32^. The area and number of mitochondria per cell were evaluated in 40 random beta cells using an image analyzer (TDI Scope Eye).

### Three-dimensional (3D) reconstruction of mitochondria

Long ribbons up to about 20 sections of isolated islets treated with drugs were cut at a thickness of 70–90 nm on an ultramicrotome (Leica Microsystems Ltd.). The area of interest was selected, and consecutive serial sections were imaged under an electron microscope (JEM 1010; JEOL, Tokyo, Japan). Outlines of individual mitochondria were manually traced with different colors through image stacks using the Photoshop software (Adobe Systems, San Jose, CA, USA). Each image was aligned, and the marked structures were masked and exported to the 3D modeling program Mimics v.19.0 (Materialise, Leuven, Belgium), along with information regarding slice thickness, actual pixel size, and image orientation. The mitochondrial volume and surface area in a stack consisting of equal numbers of sections were also calculated using Mimics v.19.0.

### Cell culture

INS-1 (rat insulinoma cell line) was grown in conditioned RPMI-1640 medium in a humidified atmosphere containing 5% CO_2_. Cells were plated in culture dishes and treated with Tac (50 μg/ml, diluted in DMSO) and CoQ_10_ (1 pg/ml–10 μg/ml, diluted in DMSO) for 12 h.

### Cell viability and apoptosis

For assessment of cell viability, INS-1 cells were plated in culture dishes at 90% confluence. On the following day, the cells were treated with Tac (50 µg/ml) and 1 pg/ml–10 μg/ml CoQ_10_ for 12 h. CCK-8 solution (Dojindo, Rockville, MD, USA) was added to each well to evaluate cell viability. For assessment of apoptosis, the cells detached by trypsin were treated with annexin V (BD Biosciences, San Jose, CA, USA) in binding buffer (BD Biosciences). The cells were analyzed using a FACSCalibur flow cytometer (BD Biosciences). Data was reported as the percentage of fluorescent cells compared to the total cell number.

### Measurement of mitochondrial ROS

The cells were treated with MitoSOX Red (Invitrogen) for 30 min at 37 °C to detect mitochondrial ROS (superoxide anion) levels using a FACSCalibur flow cytometer (BD Biosciences). Side and forward scatter data were obtained (10,000 events per sample).

### Live cell time-lapse imaging

INS-1 cells were cultured in a 12-well plate and then incubated with 1 μM of MitoSOX Red (Invitrogen) and drugs. Automated live-cell imaging was performed on a Lionheart FX Automated Microscope (BioTeck Instruments Inc., Winooski, VT, USA) with attached plate handler and live-cell incubator using a 10 × objective. Fluorescence images were captured every 1 h for 12 h, with optical emission and excitation wavelengths of 580 nm and 510 nm, respectively.

### Oxygen consumption rate (OCR) experiments

Real-time OCR in the cells was evaluated using an XF24 Extracellular Flux Analyzer (Seahorse, Billerica, MA, USA). In a non-CO_2_ incubator, the medium of the cells was changed to a running medium following incubation with drugs or Vh and treated for 1 h at 37 °C. The mitochondrial inhibitors used were 1 µM of oligomycin (ATP synthase inhibitor), 0.5 µM of carbonyl cyanide-4 (trifluoromethoxy) phenylhydrazone (FCCP), and 0.5 µM of rotenone/antimycin A (complex I and III inhibitor). Immediately following completion of the assay, each well was washed with phosphate-buffered saline, and lysis buffer (Pierce Biotechnology, Rockford, IL, USA) was added to lyse the cells in the well. Total protein was measured using the BCA Protein Assay Kit (Pierce Biotechnology). Then, the OCR data were normalized to the total protein content of each sample. Parameters for mitochondrial function were evaluated by measuring these mitochondrial inhibitor compounds as modulators to determine ATP production, basal respiration, spare respiratory capacity, and maximal respiration^33^. A total of 3–4 wells were used for each group.

### Statistical analysis

The values are expressed as the mean ± standard error of at least three independent tests. Using PRISM software, multiple comparisons were carried out by one-way analysis of variance with Bonferroni’s post-hoc test (Version 7.03 for Windows, GraphPad Software, La Jolla, CA, USA). Results with P values < 0.05 were considered statistically significant.

## Results

### Effect of CoQ_10_ administration in an experimental model of Tac-induced DM

Table 1 displays alterations in the functional parameters of the experimental groups following treatment of Tac and CoQ_10_ for 28 days. The Tac + CoQ_10_ group showed larger reductions in intake-water and urine volume than that in the Tac group. Therefore, CoQ_10_ did not affect the whole-blood trough level of Tac, implying that a drug-interaction did not occur at these doses.

**Table 1:** Effect of administration of CoQ_10_ on basic parameters.

	Vh	CoQ_10_	Tac	Tac + CoQ_10_
Body weight (g)	332 ± 4	326 ± 7	288 ± 4^1,2^	317 ± 3^3^
Water intake (mL/day)	28 ± 1	22 ± 1^1^	39 ± 3^1,2^	27 ± 1^3^
Urine volume (mL/day)	14 ± 3	12 ± 1^1^	32 ± 3^1,2^	20 ± 2^3^
Trough Tac (ng/mL)	—	—	10.6 ± 0.9	10.7 ± 1.1

Vh, vehicle; CoQ_10_, coenzyme Q10; Tac, tacrolimus; Scr, serum creatinine; BUN, blood urea nitrogen. The values shown are the mean ± SE (*n* = 8). One-way ANOVA was used to analyse the data. ^1^P < 0.05 vs. Vh; ^2^P < 0.05 vs. CoQ_10_; ^3^P < 0.05 vs. Tac

**Figure 1:**
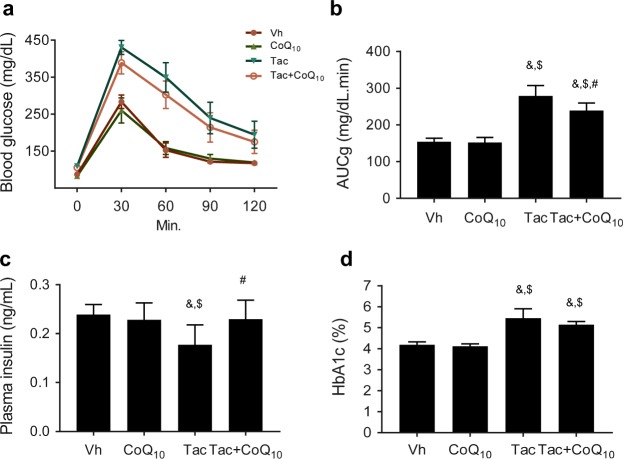
Effect of CoQ_10_ administration on Tac-induced DM in rats. Blood glucose levels were measured using IPGTTs (**a**), calculation of the AUCg from IPGTTs (**b**), plasma insulin levels (**c**), and HbA1c in each group. Data are presented as the mean ± SE (*n* = 8). One-way ANOVA was used to analyze the data. ^&^P < 0.05 versus the Vh group; ^$^P < 0.05 versus the CoQ_10_ groups; ^#^P < 0.05 versus the Tac group.

**Figure 2:**
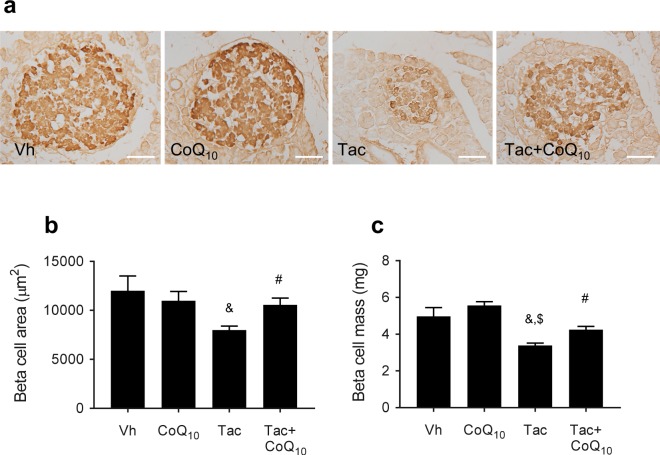
Effect of CoQ_10_ administration on pancreatic islet morphology and size during Tac-induced DM in rats. (**a**) Representative images of insulin staining in pancreatic sections. Scale bar = 50 µm (all panels). (**b**) Quantitative analysis of beta cell islet area. The Tac group showed smaller islets with a lower intensity of insulin staining within islets than the vehicle (Vh) group. In contrast, cotreatment with CoQ_10_ and Tac reversed these changes. (**c**) Quantification of estimated pancreatic beta cell mass by the point-counting method. Data are presented as the mean ± SE (*n* = 8). One-way ANOVA was used to analyze the data. ^&^P < 0.05 versus the Vh group; ^$^P < 0.05 versus the CoQ_10_ groups; ^#^P < 0.05 versus the Tac group.

Rats treated with Tac showed features of DM, as indicated by higher AUCg and HbA1c values and lower levels of plasma insulin compared with those in animals administered with Vh. Addition of CoQ_10_ reversed these changes (Fig. 1). Tac-treated islets observed lower insulin immunoreactivity and reduced numbers of beta cells than the Vh group, and these effects were reduced by treatment with CoQ_10_ (Fig. 2). We also quantified the beta cell mass in the groups and found that a reduction in beta cell mass induced by Tac treatment was reversed by cotreatment with CoQ_10_, as shown in Fig. 2c.

**Figure 3:**
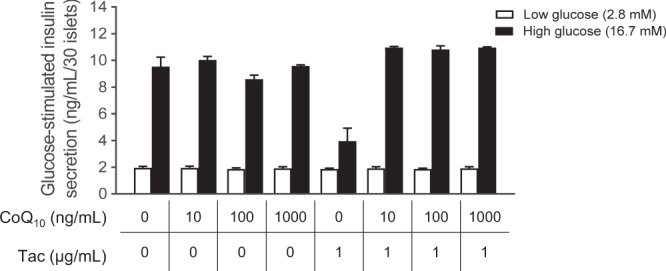
Direct effects of combined treatment with CoQ_10_ on glucose-stimulated insulin secretion (GSIS) during Tac-induced islet injury. Primary rat isolated islets were treated directly with Tac and CoQ_10_ in a culture setting. Note that all parameters recovered in the combined treatment with CoQ_10_. *n* = 8 per group. One-way ANOVA was used to analyze the data. ^&^P < 0.05 versus the other group.

To assess the exact effects of the insulin secretion function of CoQ_10_ during Tac-induced islet injury, we treated primary rat isolated islets with Tac and CoQ_10_ in a culture setting and evaluated GSIS. As expected, Tac significantly decreased GSIS. The addition of CoQ_10_ resulted in significantly higher insulin secretion levels compared with that of Tac alone, as shown in Fig. 3.

### Effect of CoQ_10_ administration on Tac-induced oxidative stress and apoptosis in pancreatic beta cells

**Figure 4:**
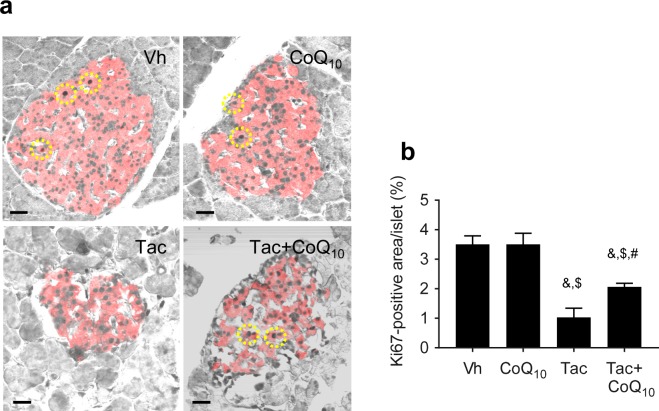
Effect of CoQ_10_ on Ki67 expression during Tac-induced islet injury. Representative images (**a**) and quantification (**b**) of double immunohistochemistry for Ki67 (gray color, marker for cell proliferation) and insulin (red fluorescence, marker for beta cells) in tissue sections from rats. Yellow dotted circles indicate Ki67-positive cells. Scale bar = 20 µm (all panels). Data are presented as the mean ± SE (*n* = 8). One-way ANOVA was used to analyze the data. ^&^P < 0.05 versus the Vh group; ^$^P < 0.05 versus the CoQ_10_ groups; ^#^P < 0.05 versus the Tac group.

**Figure 5:**
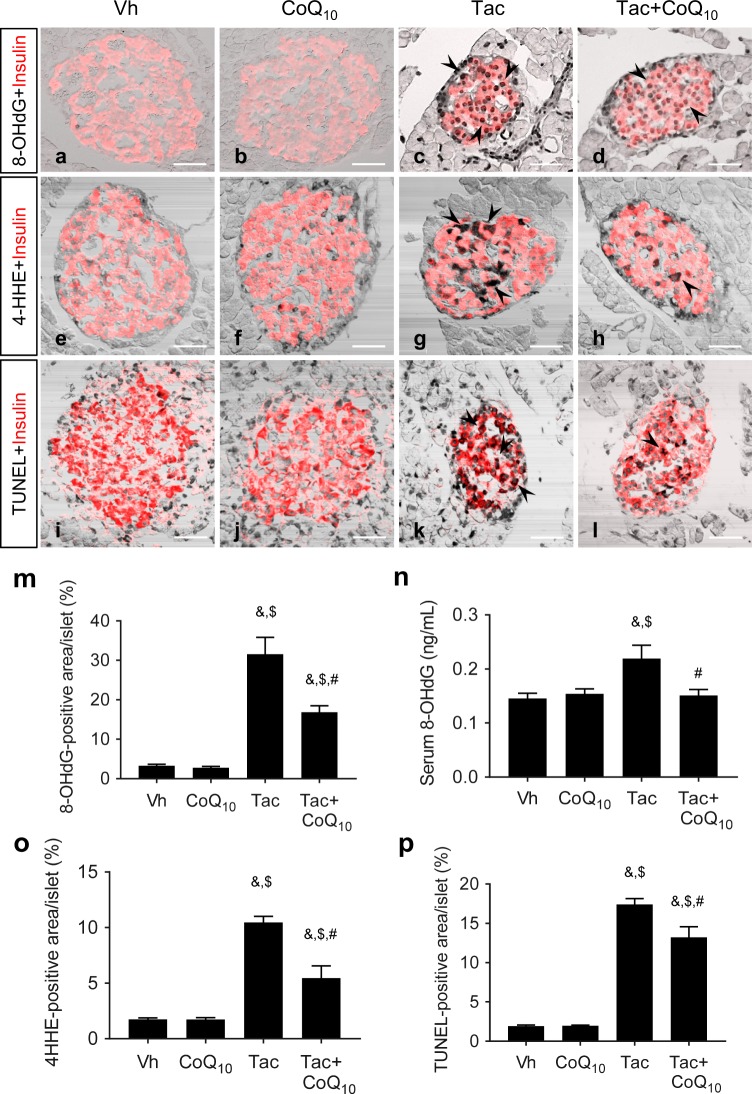
Effects of CoQ_10_ on oxidative stress and apoptosis in pancreatic islets and sera during Tac-induced DM in rats. Representative images and quantification of immunohistochemistry for 8-OHdG (**a**–**d**,**m**) and 4-HHE (**e**–**h**,**o**) and TUNEL assays (**i**–**l**,**p**) using tissue sections from rats. (**n**) Serum 8-OHdG excretion. Arrowheads indicate 8-OHdG-, 4-HHE-, and TUNEL-positive cells. Scale bar = 50 µm (all panels). Data are presented as the mean ± SE (*n* = 8). One-way ANOVA was used to analyze the data. ^&^P < 0.05 versus the Vh group; ^$^P < 0.05 versus the CoQ_10_ groups; ^#^P < 0.05 versus the Tac group.

The proliferation of beta cells was confirmed by tissue expression of Ki67 from the experimental groups (Fig. 4). Treatment of Tac reduced the number of Ki67-positive cells in the insulin-positive area compared with those in the Vh and CoQ_10_ groups, while concomitant CoQ_10_ treatment restored Ki67 staining. Figure 5 shows the co-immunohistochemical staining results for 8-OHdG (Fig. 5a–d) and 4-HHE (Fig. 5e–h) with insulin (red fluorescence), as well as levels of serum 8-OHdG (Fig. 5n). The immunoreactivity of 8-OHdG and 4-HHE in tissue sections and the serum 8-OHdG level were significantly increased in the Tac group, and these effects were restored by CoQ_10_ treatment. We also examined whether CoQ_10_ protects against apoptosis by Tac treatment. The number of TUNEL-positive cells were markedly higher in the Tac group than in the Vh group and were decreased with the cotreatment of CoQ_10_ (Fig. 5i–l,p).

### Effect of CoQ_10_ administration on Tac-induced mitochondrial ultrastructural changes in pancreatic beta cells

**Figure 6:**
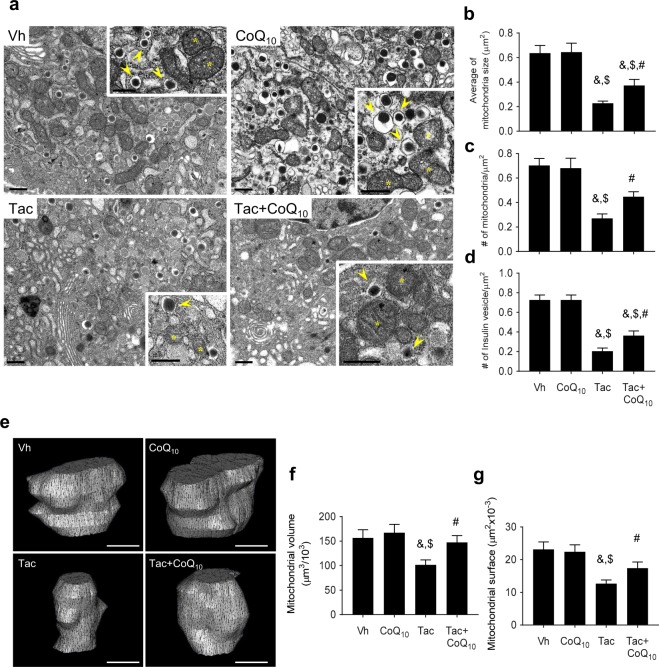
Transmission electron microscopy. (**a**) Mitochondrial ultrastructure in pancreatic beta cells. White asterisks indicate mitochondria. Arrowheads indicate insulin granules. Scale bar = 0.5 µm (panels in a). (**b**–**d**) Quantitative analysis of mitochondrial area and number of insulin granules in the experimental groups. (**e**–**g**) Three-dimensional (3D) reconstruction of mitochondria in isolated islets treated with drugs. (**e**) Representative 3D reconstruction of mitochondria, revealing mitochondrial morphology. The reconstruction was based on a series of about 20 sections cut using an ultramicrotome. Scale bar = 200 nm (panels in e). Quantitative analysis of mitochondrial volume (**f**) and surface area (**g**). Data are presented as the mean ± SE (*n* = 8). One-way ANOVA was used to analyze the data. ^&^P < 0.05 versus the Vh group; ^$^P < 0.05 versus the CoQ_10_ groups; ^#^P < 0.05 versus the Tac group.

To evaluate mitochondrial function, electron microscopy was used to assess the mitochondrial ultrastructure and quantify the mitochondrial number. For the islet cells obtained from the current experimental rats, Tac treatment decreased both the average mitochondrial area and number (Fig. 6a). By contrast, the administration of CoQ_10_ recovered the average mitochondrial area and number. Electron microscopy also demonstrated that the number of insulin granules were markedly decreased by Tac treatment. CoQ_10_ was effective in reducing this attenuation in number of granules, confirming that treatment with CoQ_10_ protected islet function after Tac treatment (Fig. 6b–d). The effect of CoQ_10_ on mitochondrial volume and surface area was evaluated by performing 3D reconstruction of consecutive sections of individual mitochondria in beta cells from isolated rat islets. Consistent with the previous results, reductions in mitochondrial volume and surface area induced by Tac treatment were significantly reversed by cotreatment with CoQ_10_ (Fig. 6e,f).

### CoQ_10_ reduces Tac-induced apoptosis and mitochondrial ROS production in INS-1 cells

**Figure 7:**
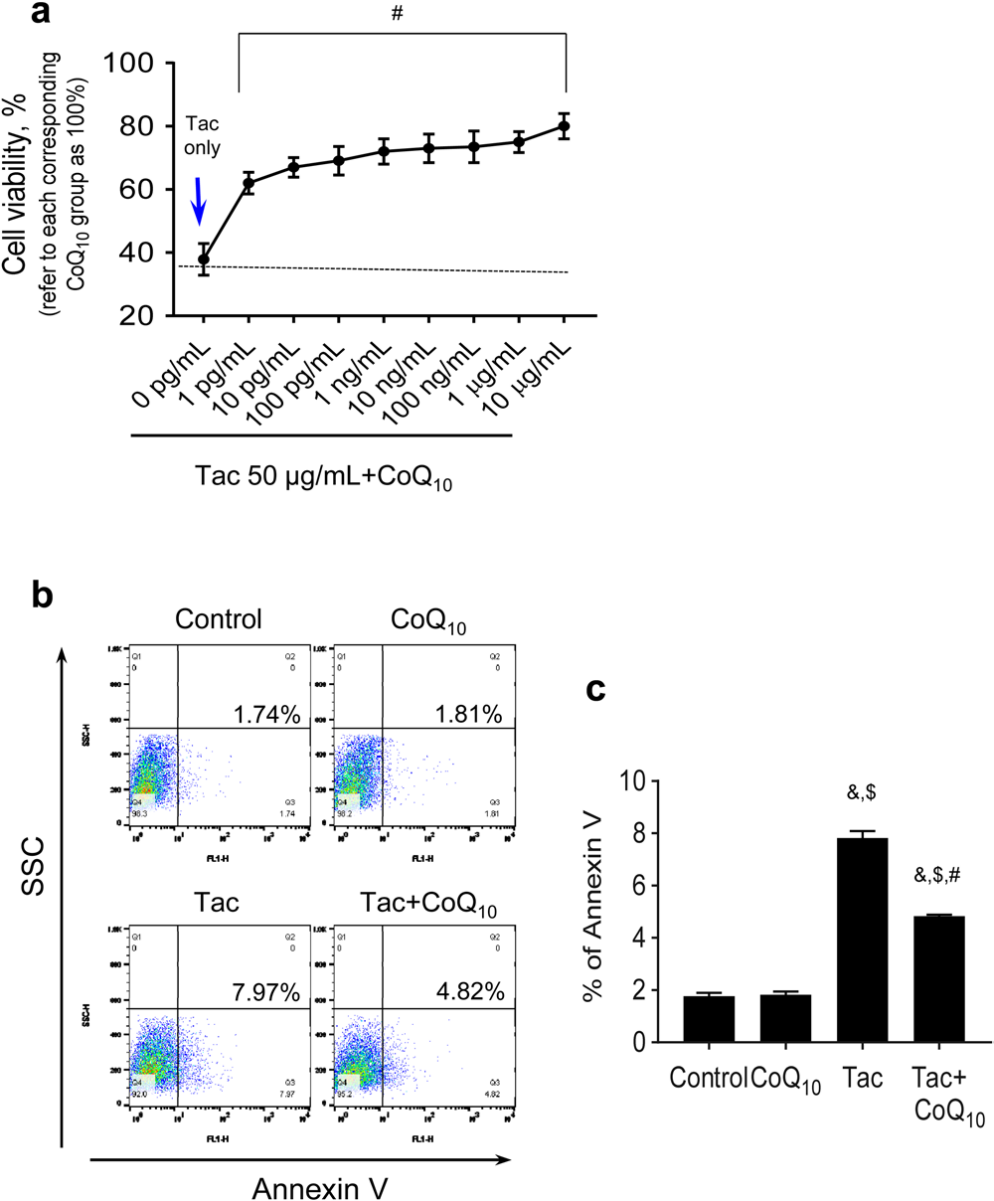
Protective effects of CoQ_10_ during Tac-induced injury in INS-1 cells. (**a**) Cell viability as assessed by CCK-8 assay. (**b** and **c**) Apoptosis as assessed by annexin V staining and flow cytometry. Data are presented as the mean ± SE (*n* = 5). One-way ANOVA was used to analyze the data. ^&^P < 0.05 versus the control group; ^$^P < 0.05 versus the CoQ_10_ groups; ^#^P < 0.05 versus the Tac group.

Using the INS-1 pancreatic beta cell line, we further evaluated the protective effect of CoQ_10_ against mitochondrial injury during Tac treatment. INS-1 cells were subjected to different concentrations (1 pg/ml–10 µg/ml) of CoQ_10_ for 12 h during Tac-induced toxicity. CoQ_10_ improved INS-1 cell viability at all doses compared with that following Tac-only treatment (Fig. 7a). To examine whether CoQ_10_ inhibits mitochondrial pathways related to apoptosis, we carried out flow cytometric analysis with annexin V staining. The Tac-induced increase in the percentage of annexin V-positive cells was significantly attenuated by CoQ_10_ treatment (Fig. 7b,c).

**Figure 8:**
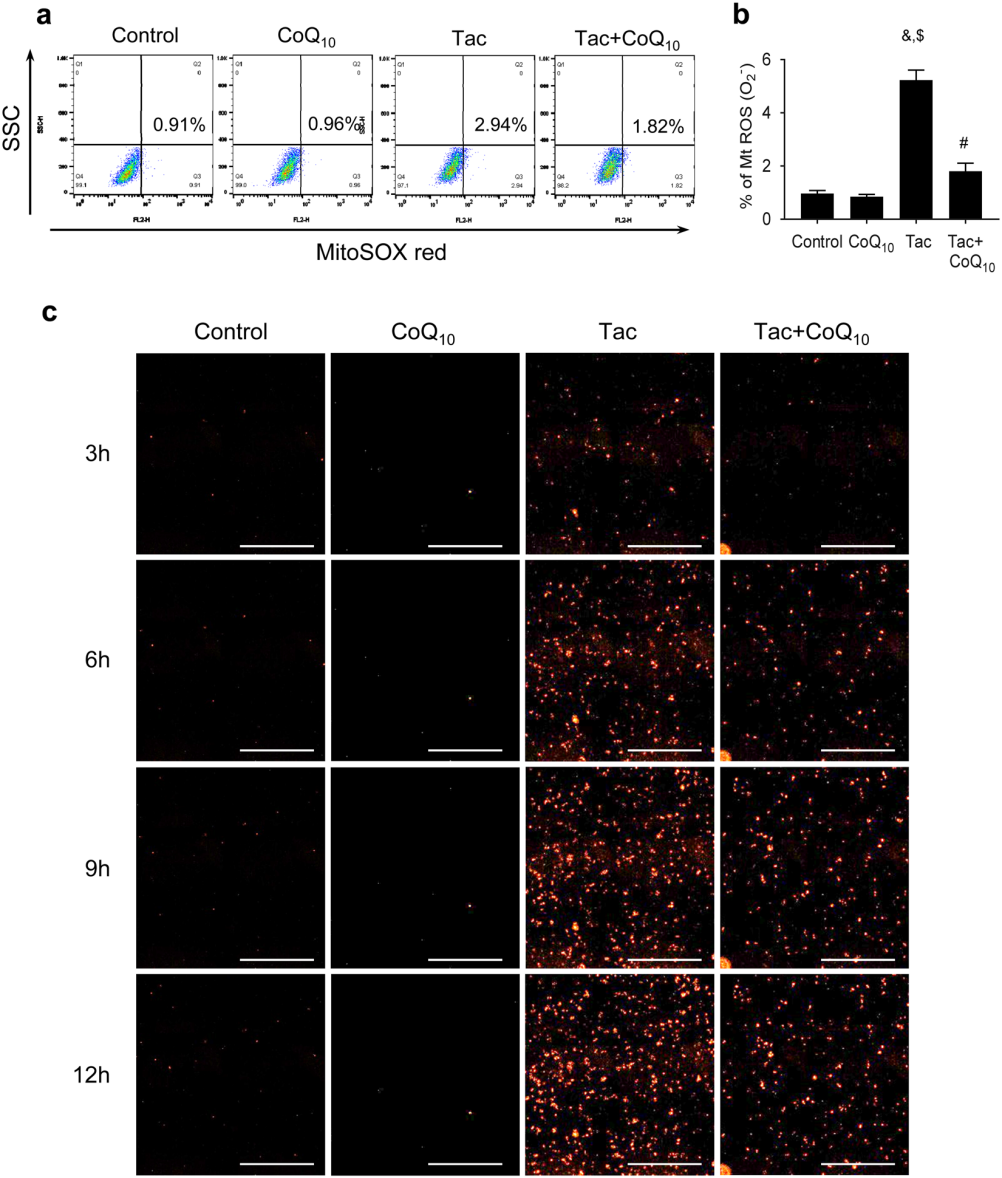
Effects of CoQ_10_ during Tac-induced mitochondrial ROS production in INS-1 cells. (**a**,**b**) MitoSOX Red staining to detect mitochondrial superoxide anion (O_2_^−^) levels by flow cytometric analysis. (**c**) Representative time-lapse images of cells treated for 12 h with MitoSOX Red in the presence or absence of Tac and CoQ_10_ were taken from the same field. Scale bar = 1000 µm (all panels). Data are presented as the mean ± SE (*n* = 5). One-way ANOVA was used to analyze the data. ^&^P < 0.05 versus the control group; ^$^P < 0.05 versus the CoQ_10_ groups; ^#^P < 0.05 versus the Tac group.

Next, we evaluated whether treatment with CoQ_10_ reduced mitochondrial ROS accumulation during Tac treatment. Use of MitoSOX Red for mitochondrial superoxide (O_2_^−^) detection accompanied by flow cytometric analysis in INS-1 cells revealed that CoQ_10_ significantly attenuated Tac-induced MitoSOX Red fluorescence (Fig. 8a,b). To visualize the protective effect of CoQ_10_ against O_2_^−^ accumulation in mitochondria, we performed time-lapse monitoring of MitoSOX Red intensity in the presence or absence of Tac and CoQ_10_ in the same field. As shown in Fig. 8c, MitoSOX Red fluorescence intensity was dramatically elevated in the Tac group, but this effect was attenuated by CoQ_10_ treatment.

### CoQ_10_ increased mitochondrial respiration in Tac-treated INS-1 cells

**Figure 9:**
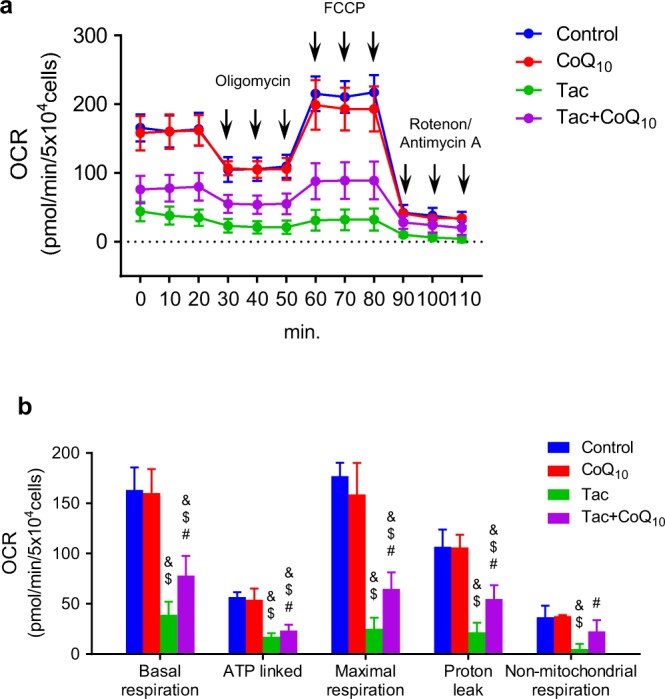
Effect of CoQ_10_ on mitochondrial function during Tac-induced injury in INS-1 cells. (**a**) The areas under the curve for basal respiration, (**b**) ATP production, maximal respiration, proton leak, and non-mitochondrial respiration were calculated from the OCR. Data are presented as the mean ± SE (*n* = 5). One-way ANOVA was used to analyze the data. ^&^P < 0.05 versus the control group; ^$^P < 0.05 versus the CoQ_10_ groups; ^#^P < 0.05 versus the Tac group.

The mitochondrial bioenergetics of whole cells was determined by calculating oxygen consumption over time after the sequential addition of inhibitors of mitochondrial function. This analysis revealed marked differences between the Tac and Tac + CoQ_10_ groups (Fig. 9a). Compared to Tac-only treatment, Tac + CoQ_10_ treatment resulted in higher rates of basal mitochondrial respiration, which is usually indicative of either a higher number of mitochondria or increased mitochondrial activity (Fig. 7b). Moreover, compared with Tac-only treatment, Tac + CoQ_10_ treatment resulted in significantly higher ATP-linked respiration and maximal respiration.

## Discussion

**Figure 10:**
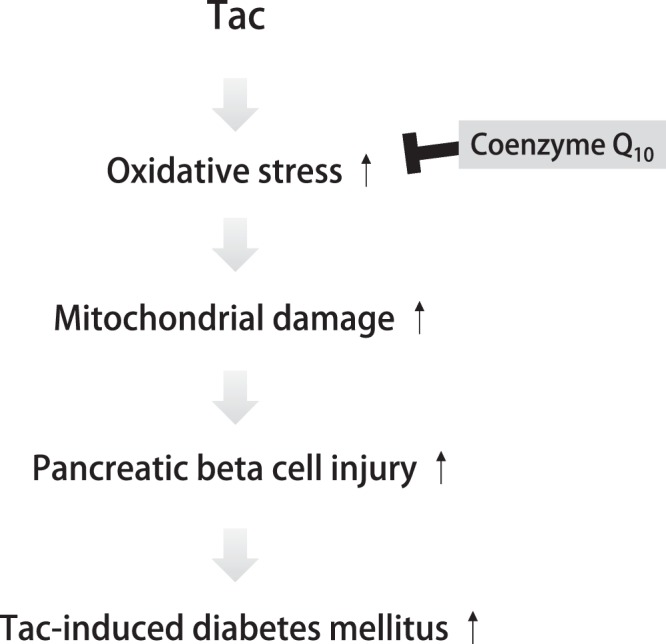
Proposed mechanism of the protective effect of CoQ_10_ during Tac-induced pancreatic beta cell injury.

The current study was performed to investigate whether cotreatment with CoQ_10_ is effective in ameliorating pancreatic beta cell dysfunction by Tac. The results of our study showed that CoQ_10_ attenuated hyperglycemia and restored the insulin secretion ability by reducing Tac-induced oxidative stress. These findings suggest that CoQ_10_ produces beneficial effects for reducing mitochondrial injury via its antioxidative properties during Tac-induced beta cell injury (Fig. 10). The results of our study thus provide a rationale for use of CoQ_10_ as supplemental therapy in Tac-induced DM in clinical practice.

In this study, we first evaluated whether the administration of CoQ_10_ was effective in controlling hyperglycemia in an experimental Tac-induced DM model. Recently, several clinical studies have shown that CoQ_10_ treatment reduces DM^34–36^. In this study, we found that the AUCg based on IPGTT was increased and the plasma insulin level and insulin-immunoreactivity in islets were decreased by chronic Tac treatment. However, cotreatment with CoQ_10_ significantly decreased the AUCg and increased levels of plasma insulin and GSIS compared with the levels observed following Tac treatment alone. Furthermore, immunostaining of insulin in islets revealed that concomitant treatment with CoQ_10_ resulted in increases in islet size and immunoreactivity for insulin, as well as preservation of islet morphology as demonstrated by less irregular islet boundaries and reduced vacuolization. These findings suggest that CoQ_10_ has beneficial effects on the preservation of beta cells during Tac treatment.

To elucidate the possible mechanism of CoQ_10_ in the control of hyperglycemia, we evaluated the expression of markers of oxidative stress in the experimental groups, as oxidative stress has been suggested to be an important pathway in the pathogenesis of pancreatic beta cell injury. High levels of oxidative stress induced by Tac administration lead to islet cell death and dysfunction, with short- and long-term Tac treatment inducing the production of ROS, thereby causing apoptotic cell death^32,37^. Therefore, it has been proposed that a reduction in oxidative stress could protect from pancreatic beta cell injury. Based on this hypothesis, we focused on the antioxidative effect of CoQ_10_ on Tac-induced oxidative injury in pancreatic beta cells. For this, we measured the expression of 8-OHdG and 4-HHE, markers of oxidative DNA and lipid damage, respectively, and found that while their expression levels were increased in beta cells in Tac-treated mice, administration of CoQ_10_ attenuated these increases in expression levels. Indeed, there is evidence to show that the development of diabetes is associated with increased oxidative stress and that CoQ_10_ can scavenge ROS, resulting in an anti-hyperglycemic effect^25^. Overall, our data indicate that oral administration of CoQ_10_ is effective in reducing Tac-induced oxidative stress in pancreatic beta cells.

We further evaluated whether the antioxidative effect of CoQ_10_ ameliorated mitochondrial injury during Tac treatment. The mitochondria play important roles in pancreatic beta cells from glucose metabolism to insulin exocytosis, thereby ensuring tight regulation of glucose-stimulated insulin secretion^38^. Impairment in the mitochondrial function affects this metabolic coupling and ultimately leads to apoptosis and beta cell death. Furthermore, mitochondria are susceptible to oxidative damage and could be a major source of superoxide under disease conditions^39^. Based on this knowledge, we first examined rates of apoptosis, a mitochondrial pathway of cell death *in vivo* and *in vitro*, and found that CoQ_10_ reduced the numbers of TUNEL- and annexin V-positive cells compared with those in the Tac group. Ultrastructural analysis revealed that Tac treatment caused reductions in the number, area, size, and volume of mitochondria, as well as the number of insulin granules, while CoQ_10_ administration attenuated these changes. CoQ_10_ also reduced accumulation of mitochondrial ROS and superoxide anion and restored basal respiration, ATP-linked respiration, and maximal respiration rates according to oxygen consumption over time. These data suggest that administration of CoQ_10_ helps to maintain mitochondrial function during Tac-induced oxidative stress, resulting in a subsequent decrease in apoptotic cell death.

In addition to Tac, rapamycin analogs, sirolimus (SRL) and everolimus (EVR), have been widely used as immunosuppressants in transplantation, but they are also associated with an increased risk of DM. Many studies have demonstrated that both of these drugs cause mitochondrial dysfunction in pancreatic beta cells, eventually leading to decreased insulin release^40–43^. We have also confirmed that SRL or EVR treatment alone results in the development of hyperglycemia, accompanied by a reduction in the number of insulin granules and an increase in the expression of oxidative stress markers, in animal models^27,30,31^. However, addition of CoQ_10_ attenuated SRL-induced hyperglycemia, as well as oxidative stress, in a rat model. At the subcellular level, CoQ_10_ also improved not only the morphology of the mitochondria but also mitochondrial respiration^25^. Based on these findings, CoQ_10_ supplementation would also be beneficial in the treatment of rapamycin-induced DM.

In conclusion, CoQ_10_ plays an important role in reducing Tac-induced oxidative stress and protecting mitochondria in pancreatic beta cells. These findings suggest that CoQ_10_ may be useful in the management of Tac-induced DM in the future.

## References

[1] Kasiske BL, Snyder JJ, Gilbertson D, Matas AJ (2003). Diabetes mellitus after kidney transplantation in the United States. Am J Transplant.

[2] Weir M (2001). Impact of immunosuppressive regimes on posttransplant diabetes mellitus. Transplant Proc.

[3] Heit JJ (2006). Calcineurin/NFAT signalling regulates pancreatic beta-cell growth and function. Nature.

[4] Soleimanpour SA (2010). Calcineurin signaling regulates human islet {beta}-cell survival. J Biol Chem.

[5] Demozay D, Tsunekawa S, Briaud I, Shah R, Rhodes CJ (2011). Specific glucose-induced control of insulin receptor substrate-2 expression is mediated via Ca2+-dependent calcineurin/NFAT signaling in primary pancreatic islet beta-cells. Diabetes.

[6] Yang J (2009). PANDER binds to the liver cell membrane and inhibits insulin signaling in HepG2 cells. FEBS Lett.

[7] Donelan MJ (2002). Ca2+-dependent dephosphorylation of kinesin heavy chain on beta-granules in pancreatic beta-cells. Implications for regulated beta-granule transport and insulin exocytosis. J Biol Chem.

[8] Lawrence MC, Bhatt HS, Watterson JM, Easom RA (2001). Regulation of insulin gene transcription by a Ca(2+)-responsive pathway involving calcineurin and nuclear factor of activated T cells. Mol Endocrinol.

[9] Redmon JB, Olson LK, Armstrong MB, Greene MJ, Robertson RP (1996). Effects of tacrolimus (FK506) on human insulin gene expression, insulin mRNA levels, and insulin secretion in HIT-T15 cells. J Clin Invest.

[10] Harbeck MC, Louie DC, Howland J, Wolf BA, Rothenberg PL (1996). Expression of insulin receptor mRNA and insulin receptor substrate 1 in pancreatic islet beta-cells. Diabetes.

[11] Ishizuka J (1993). Effects of FK506 and cyclosporine on dynamic insulin secretion from isolated dog pancreatic islets. Transplantation.

[12] Piao SG (2014). Combined treatment of tacrolimus and everolimus increases oxidative stress by pharmacological interactions. Transplantation.

[13] Trinanes J (2017). Deciphering Tacrolimus-Induced Toxicity in Pancreatic beta Cells. Am J Transplant.

[14] Lenaz G, Fato R, Formiggini G, Genova ML (2007). The role of Coenzyme Q in mitochondrial electron transport. Mitochondrion.

[15] Crane FL (2001). Biochemical functions of coenzyme Q10. J Am Coll Nutr.

[16] Bentinger M, Brismar K, Dallner G (2007). The antioxidant role of coenzyme Q. Mitochondrion.

[17] Huo J (2018). Coenzyme Q10 Prevents Senescence and Dysfunction Caused by Oxidative Stress in Vascular Endothelial Cells. Oxid Med Cell Longev.

[18] Venegoni W (2017). The use of antioxidants in the treatment of traumatic brain injury. J Adv Nurs.

[19] Ishikawa, A. & Homma, Y. Beneficial effect of ubiquinol, the reduced form of coenzyme Q10, on cyclosporine nephrotoxicity. *Int Braz J Urol* **38**, 230–234; discussion 234 (2012). 10.1590/s1677-55382012000200011 22555041

[20] Chen H, Tappel AL (1995). Vitamin E, selenium, trolox C, ascorbic acid palmitate, acetylcysteine, coenzyme Q, beta-carotene, canthaxanthin, and (+)-catechin protect against oxidative damage to kidney, heart, lung and spleen. Free Radic Res.

[21] Upaganlawar A, Farswan M, Rathod S, Balaraman R (2006). Modification of biochemical parameters of gentamicin nephrotoxicity by coenzyme Q10 and green tea in rats. Indian J Exp Biol.

[22] Sohet FM (2009). Coenzyme Q10 supplementation lowers hepatic oxidative stress and inflammation associated with diet-induced obesity in mice. Biochem Pharmacol.

[23] Spindler M, Beal MF, Henchcliffe C (2009). Coenzyme Q10 effects in neurodegenerative disease. Neuropsychiatr Dis Treat.

[24] Lombardi A, Trimarco B, Iaccarino G, Santulli G (2017). Impaired mitochondrial calcium uptake caused by tacrolimus underlies beta-cell failure. Cell Commun Signal.

[25] Sun, I. O. *et al*. The effects of addition of coenzyme Q10 to metformin on sirolimus-induced diabetes mellitus. *Korean J Intern Med* (2017). 10.3904/kjim.2017.004 PMC6406080 29228766

[26] Jin J (2017). Effect of Empagliflozin on Tacrolimus-Induced Pancreas Islet Dysfunction and Renal Injury. Am J Transplant.

[27] Piao SG (2012). Drug interaction between cyclosporine and mTOR inhibitors in experimental model of chronic cyclosporine nephrotoxicity and pancreatic islet dysfunction. Transplantation.

[28] ER, W. *Stereologkal methods vol 1*. Academic Press Inc.: London, 1979.

[29] Bonner-Weir S (2001). Beta-cell turnover: its assessment and implications. Diabetes.

[30] Jin J (2017). Effects of metformin on hyperglycemia in an experimental model of tacrolimus- and sirolimus-induced diabetic rats. Korean J Intern Med.

[31] Jin L, Lim SW, Jin J, Chung BH, Yang CW (2016). Effects of addition of a dipeptidyl peptidase IV inhibitor to metformin on sirolimus-induced diabetes mellitus. Transl Res.

[32] Lim SW, Jin L, Luo K, Jin J, Yang CW (2017). Ginseng extract reduces tacrolimus-induced oxidative stress by modulating autophagy in pancreatic beta cells. Lab Invest.

[33] Dott W, Mistry P, Wright J, Cain K, Herbert KE (2014). Modulation of mitochondrial bioenergetics in a skeletal muscle cell line model of mitochondrial toxicity. Redox Biol.

[34] Yen CH, Chu YJ, Lee BJ, Lin YC, Lin PT (2018). Effect of liquid ubiquinol supplementation on glucose, lipids and antioxidant capacity in type 2 diabetes patients: a double-blind, randomised, placebo-controlled trial. Br J Nutr.

[35] Gholami, M., Zarei, P., Sadeghi Sedeh, B., Rafiei, F. & Khosrowbeygi, A. Effects of coenzyme Q10 supplementation on serum values of adiponectin, leptin, 8-isoprostane and malondialdehyde in women with type 2 diabetes. *Gynecol Endocrinol*, 1–5 (2018). 10.1080/09513590.2018.1481944 29933718

[36] Stojanovic M, Radenkovic M (2017). A meta-analysis of randomized and placebo-controlled clinical trials suggests that coenzyme Q10 at low dose improves glucose and HbA1c levels. Nutr Res.

[37] Lim SW, Jin L, Jin J, Yang CW (2016). Effect of Exendin-4 on Autophagy Clearance in Beta Cell of Rats with Tacrolimus-induced Diabetes Mellitus. Sci Rep.

[38] Supale S, Li N, Brun T, Maechler P (2012). Mitochondrial dysfunction in pancreatic beta cells. Trends Endocrinol Metab.

[39] Smith RA (2008). Mitochondria-targeted antioxidants in the treatment of disease. Ann N Y Acad Sci.

[40] Suzuki L (2018). Everolimus Directly Suppresses Insulin Secretion Independently of Cell Growth Inhibition. J Endocr Soc.

[41] Manzia TM (2018). Ab initio Everolimus-based Versus Standard Calcineurin Inhibitor Immunosuppression Regimen in Liver Transplant Recipients. Transplant Proc.

[42] Lombardi A (2017). Sirolimus induces depletion of intracellular calcium stores and mitochondrial dysfunction in pancreatic beta cells. Sci Rep.

[43] Constantinescu AA (2016). Differential influence of tacrolimus and sirolimus on mitochondrial-dependent signaling for apoptosis in pancreatic cells. Mol Cell Biochem.

